# Intracellular Ca^2+^ Signaling in Protozoan Parasites: An Overview with a Focus on Mitochondria

**DOI:** 10.3390/ijms22010469

**Published:** 2021-01-05

**Authors:** Pedro H. Scarpelli, Mateus F. Pecenin, Celia R. S. Garcia

**Affiliations:** Department of Clinical and Toxicological Analyses, School of Pharmaceutical Sciences, University of São Paulo, Avenida Professor Lineu Prestes, 580, bloco 17, São Paulo 05508-000, Brazil; pedroscarpelli2@gmail.com (P.H.S.); filap.mateus@gmail.com (M.F.P.)

**Keywords:** mitochondria, calcium signaling, protozoan parasites

## Abstract

Ca^2+^ signaling has been involved in controling critical cellular functions such as activation of proteases, cell death, and cell cycle control. The endoplasmatic reticulum plays a significant role in Ca^2+^ storage inside the cell, but mitochondria have long been recognized as a fundamental Ca^2+^ pool. Protozoan parasites such as *Plasmodium falciparum*, *Toxoplasma gondii*, and *Trypanosoma cruzi* display a Ca^2+^ signaling toolkit with similarities to higher eukaryotes, including the participation of mitochondria in Ca^2+^-dependent signaling events. This review summarizes the most recent knowledge in mitochondrial Ca^2+^ signaling in protozoan parasites, focusing on the mechanism involved in mitochondrial Ca^2+^ uptake by pathogenic protists.

## 1. Calcium Channels, Receptors, Compartmentalization, and Signaling in Animal Cells

Evolutionarily, Ca^2+^ ions have emerged as one of the most important second messengers that regulate different cellular processes, from muscle contractions and synapses to cell division and apoptosis [1,2]. Therefore, the precise regulation of this ion is a common feature among all forms of life. Cooperation between channels, transport pumps, and stock organelles promote homeostasis of Ca^2+^ ions, preventing cytotoxicity, and the consequent cell death caused by uncontrolled ion increase [3].

Apart from their role in energy metabolism, mitochondria also participate in the Ca^2+^ signaling inside the cell, reaching micromolar values of Ca^2+^. Ca^2+^ accumulation into the mitochondria stimulates ATP production by modulating enzymes of the tricarboxylic acid cycle (TCA cycle) such as pyruvate dehydrogenase, 2-oxoglutarate, and isocitrate-dehydrogenases. Additionally, a high concentration of Ca^2+^ inside the cell may trigger cell death by resulting in an excessive mitochondrial Ca^2+^ uptake and the release of apoptotic factors [4].

The transport of Ca^2+^ into and out of the mitochondria is tightly regulated by channels and transporters located in the outer and inner mitochondrial membrane. The increase in cytoplasmatic (Ca^2+^) occurs through two mechanisms in eukaryotes: the exit of Ca^2+^ from intracellular stores to the cytoplasm, and the entry of Ca^2+^ through the cell membrane [5]. Ca^2+^ permeable channels, such as voltage Ca^2+^ channels (VGCC) and P2X ionotropic receptors, participate in the increase of Ca^2+^ influx through the plasma membrane in excitable cells. In non-excitable cells, the mobilization of intracellular stores guarantees Ca^2+^ dependent cell signaling. In addition to membrane channels, ATP-dependent pumps and Na^+^/Ca^2+^ exchangers promote the cell’s Ca^2+^ efflux, maintaining the low intracellular concentration.

In excitable cells, changes in ion gradients (Ca^2+^, K^+^, Cl^−^, Na^+^) through the plasma membrane’s depolarization regulate essential activities that generate physiological responses, such as muscle contraction, neurotransmitter secretion, learning, and memory mechanisms. Voltage-operated Ca^2+^ channels are examples of proteins present on the plasma membrane’s surface that regulate ion concentration in the cytoplasm, generating signaling and cellular response [6,7,8]. These channels transform the electrical excitability from membrane depolarization into cell signaling mediated by an increase in cytoplasmatic (Ca^2+^).

Ionotropic receptors are ion channels also present in the cell membrane that, when activated by an agonist, allow the passage of ions to the cell’s cytosol. An example is the P2X family purinoceptors. These receptors are present in different excitable and non-excitable cells and when activated by ATP, have high permeability to monovalent cations, Ca^2+,^ and other anions [9,10].

The transient receptor potential (TRP) ion channels are membrane proteins found in several tissues and cell types, permeable to mono- or divalent cations, and are involved in cellular responses such as the perception of stimuli (temperature, pheromones, pain, taste) and ion homeostasis [11,12,13]. The stimulation of TRP channels promotes cellular depolarization with the consequent activation of voltage-gated ion channels. In addition, Ca^2+^ permeable TRP channels regulate intracellular ion concentration, and therefore different cellular responses [11].

One of the most extensive stocks of Ca^2+^ in animal cells is the endoplasmic reticulum (ER). Two ion channels present in the ER membrane are responsible for the release of Ca^2+^ ions from the organelle to the cytosol: the inositol 1,4,5-triphosphate receptor (IP3R) and the ryanodine receptor (RyR). IP3R activation occurs through a signaling pathway mediated by second messengers that involve the activation of G protein-coupled receptors (GPCRs) [14,15]. Briefly, in response to the stimulation of GPCRs, phospholipase C (PLC) catalyzes the transformation of phosphoinositol-4,5-bisphosphate (PIP2) into IP3 and diacylglycerol (DAG). IP3 binds to its receptor, activating it, depleting Ca^2+^ from the ER, and increasing cytoplasmatic (Ca^2+^). Three types of IP3R are described in vertebrates, IP3R types 1–3, and differences in splicing and phosphorylation sites. In affinity for IP3 and associated molecules, they promote unique responses attributed to each different destination of the activated signaling pathways [16].

Another channel that regulates the output of Ca^2+^ to the cytosol is RyR. This receptor can be activated indirectly by stimulating the voltage modulated channel type-L Cav1.1–1.2, and by Ca^2+^, Mg^2+^ ions, protein kinase A (PKA), calmodulin (CaM), Ca^2+^ dependent protein kinase/calmodulin (CaMK), FK506 binding proteins, calsequestrin (CSQ), triadin, and junction [17]. In vertebrates, three isoforms of RyR are described and homologs have already been identified in *Drosophila melanogaster*, *Caenorhabditis elegans*, and *Homarus americanus*.

The decrease in (Ca^2+^) in the ER lumen stimulates a process of interaction between Stromal interaction molecules (STIM), anchored in the ER membrane, and the Calcium release-activated calcium channel protein (ORAI) Ca^2+^ permeable channels present in the cell’s plasma membrane. This process, called store-operated calcium entry (SOCE), first described by JW Putney Jr (1986) [18], has been widely described in several types of non-excitatory cells and is fundamental for the amplification of cell signaling or to fill the intracellular Ca^2+^ stocks. Due to the high concentration of Ca^2+^ in the ER, the N-terminal domain of the STIM protein, located in the ER membrane, is linked to these ions. After the depletion of Ca^2+^ stores mediated by IP3 and consequently uncoupling of Ca^2+^ from the STIM protein, the latter dimerizes and translocates to the junction region of the ER membrane with the plasma membrane, allowing the interaction with the ORAI channels. This channel opens, allowing the passage of Ca^2+^ into the cell’s cytoplasm [19].

Cytoplasmatic Ca^2+^ is sequestered into the ER for the maintenance of intracellular Ca^2+^ stocks through an ATP-dependent protein called SERCA-ATPase, present in all eukaryotic cells [20]. This pump guarantees the low concentration of cytosolic Ca^2+^, regulating the termination of signaling pathways and preventing cell intoxication by an excess of Ca^2+^.

Acidic organelles, such as endo-lysosomes and acidocalcisomes, also contribute to cellular Ca^2+^ oscillations. Two-pore channels (TPCs) found in the membrane of endo-lysosomes of animals and plants allow the passage of Ca^2+^ ions to the cell cytoplasm when activated [21,22,23]. Acidocalcisomes are organelles first described in the parasitic protozoa *Trypanosoma brucei* [24] that maintain high internal concentrations of Ca^2+^ and are rich in orthophosphate, pyrophosphate, and polyphosphate, being conserved from bacteria to humans [25,26,27]. The internal acidity is maintained by pumps that allow the passage of hydrogen ions into the organelle’s interior. Moreover, Ca^2+^ permeable channels’ presence participates in the cytosolic Ca^2+^ oscillations, and ATP-dependent exchanger pumps transport Ca^2+^ back into the organelle, contributing to homeostasis.

Golgi Apparatus (GA) is also considered an organelle that participates in oscillations and homeostasis of Ca^2+^. Ca^2+^ permeable channels, such as IP3R, RyR, and TRPs are present in the organelle membrane and contribute to the increase in the cytoplasmatic (Ca^2+^). On the other hand, the presence of SERCA and SPCAs (secretory-pathway ATPases) guarantees the sequestration of Ca^2+^ into the GA [28,29].

Once free in the cell’s cytoplasm, Ca^2+^ can bind to different molecules and regulate different cell signaling pathways. A wide range of proteins present in other tissues has a specific motif for Ca^2+^ binding: EF-hand. Calmodulin (CaM) is an abundant and conserved protein among eukaryotes. It has the EF-hand domains and, when bound to Ca^2+^, regulates the activation of calmodulin kinase (CaMK) [30,31], which stimulates transcription and is responsible for the expression of genes and regulation of cellular functions. CaM also interacts with Calcineurin, a serine/threonine phosphatase that participates in the regulation of various cellular processes in lower and upper eukaryotes [32].

## 2. The Role of Ca^2+^ Signaling in Protozoan Parasites

The life cycle of parasitic protozoa is complex, involving multiple hosts, several cell types, different tissues, and microenvironments. This great diversity implies a finely regulated cell signaling mechanism that allows the parasites to adapt to different stimuli. For example, the *Trypanosoma cruzi* life cycle begins when a vector insect bites and releases metacyclic trypomastigotes in its feces. These forms enter the injury site and invade nearby cells, and then differentiate into intracellular amastigotes. This form can divide by binary fission and differentiate into trypomastigotes, which are released into the bloodstream. Trypomastigotes in the bloodstream can invade multiple cells, resulting in new intracellular amastigotes, or they can remain in the extracellular medium to be ingested by the insect vector. Ingested trypomastigotes differentiate into epimastigotes in the midgut of the insect vector, multiply, and differentiate into metacyclic trypomastigotes in the hindgut, ready to be released and infect a new host and continue the cycle. In parallel, the *Plasmodium falciparum* cycle is more complex: it begins with the bite of an infected anopheles mosquito, which injects sporozoites into the host. Once in the bloodstream, sporozoites migrate to the liver and invade hepatocytes, where they can remain inactive or replicate asexually, forming a large number of merozoites in the host cell. The release of merozoites into the bloodstream marks the beginning of the erythrocytic stages. Merozoites invade erythrocytes and develop within the parasitophorous vacuole, undergoing various biochemical and morphological transformations, which can be identified by three stages called ring, trophozoite, and schizont. The erythrocyte rupture by schizonts releases new merozoites, continuing the intra-erythrocytic cycle. During the cycle, a small percentage of parasites differentiate in female or male gametocytes, capable of infecting the vector mosquito during blood ingestion. In the mosquito’s intestine, gametocytes mature in macrogametocyte (female) and exflagellated microgametocyte (male), which is followed by fertilization and zygote formation. The zygote migrates to the intestinal epithelium, where it develops into an oocyst. The rupture of the oocyst releases sporozoites, which migrate to the salivary gland and are injected into the human bloodstream during the mosquito’s feeding, completing the cycle.

Intracellular Ca^2+^ signaling is essential for cell mobility, invasion, and egress of the host cell and cell differentiation in some protozoa. Among them, the ciliates (*Paramecium* spp.), Trypanosomatids (*Trypanosoma* spp., *Leishmania* spp.), and apicomplexan *Plasmodium* spp., *Toxoplasma* spp., *Cryptosporidium* spp., *Babesia* spp.) [33,34]. Due to the significant evolutionary distance between some eukaryotic model organisms, such as *Paramecium* and Apicomplexa, little is known about the mechanisms that regulate calcium-mediated concentration and signaling in these organisms [35,36].

The free-living ciliated protozoan *Paramecium* spp. is a model organism that has been widely studied on the mechanisms of intracellular (Ca^2+^) regulation. A range of Ca^2+^ permeable channels have already been described in these ciliates. These include IP3R and RyR homologs present in different cell compartments, ATP-dependent channels (SERCA and PMCA), voltage-operated, and mechanosensitive channels. Ca^2+^ binding proteins, such as calmodulins, calcineurins, and protein kinases, have also been identified [34,37,38,39,40].

Trypanosomatids are a large group of protozoa that include two genera of great importance for human health: *Trypanosoma* and *Leishmania*. Several Ca^2+^ permeable channels have been described in *Trypanosoma* spp. and *Leishmania* spp. among them: Ca^2+^-ATPase channels SERCA homologs are present in the ER; Ca^2+^-ATPases (PMCA) present in the plasma membrane and acidocalcisomes; voltage-operated channels present in the plasma membrane; IP3R homolog channels present in acidocalcisomes; TRP channels present in acid organelles; and Ca^2+^ influx and efflux channels present in the mitochondria. Ca^2+^ binding proteins, such as CaM, CaM-like proteins, calcireticulin, and Ca^2+^ binding proteins present in the flagellum membrane were also identified [41,42,43].

In *Plasmodium*, *Toxoplasma,* and *Cryptosporidium* organisms, some Ca^2+^-ATPases proteins are known, such as SERCA in the ER, Ca^2+^ transporters in the GA membrane, Ca^2+^/H^+^ exchanger pumps, and components that regulate upstream signaling via Ca^2+^ [33,36,44,45,46]. In *Toxoplasma gondii,* TPC and TRPs homologous Ca^2+^ channels were identified, but none of the three organisms shows a canonic mitochondrial calcium uniporter (MCU)-type channel [43,47]. Pharmacological evidence in *Plasmodium* and *Toxoplasma* indicate that the mobilization of Ca^2+^ from intracellular stocks occurs via the PLC-IP3 pathway [34,48] and by cyclic ADP ribose [49], respectively. In addition, *P. falciparum* parasites display four GPCR-like proteins [50] and one of them, PfSR25, is enrolled in Ca^2+^ and PLC signaling [51]. However, there is no confirmation of the presence of IP3R or RyR in Apicomplexa. The genomic analysis identified Ca^2+^ binding proteins in apicomplexan parasites, such as calmodulins and most notably calcium-dependent protein kinases (CDPKs) [33,36,44,52].

In *Plasmodium*, ER is one of the main intracellular stores of Ca^2+^, in addition to the mitochondria and an acidocalcisome [52,53,54]. Pereira et al. (2020) [55] recently reported a newly generated transgenic line of *P. falciparum* (PfGCaMP3) that expresses the genetically encoded Ca^2+^ indicator GCaMP3. The authors showed the dynamics of Ca^2+^ release and influx elicited by inhibitors of the SERCA pumps, cyclopiazonic acid (CPA), and Thapsigargin (Thg) [55]. Only one canonical sequence of the SERCA Ca^2+^-ATPase (PfATP6) transporter was identified in the *P. falciparum* genome [33].

Oscillations in the cytoplasmatic (Ca^2+^) are essential for the hepatic stage of the malaria parasite, thus activating motility, regulating the secretion of adhesion and invasion proteins sporozoites. Carey et al. (2014) demonstrated that sporozoites treated with a Ca^2+^ chelating agent, PLC inhibitor, or IP3R inhibitor harmed motility and adhesin secretion, highlighting the importance of the PLC-IP3 pathway during the parasite’s liver cycle [56]. By using a protein knockdown system, Philip and Waters (2015) [57] induced depletion of calcineurin. They observed a reduction in *P. berghei* sporozoites’ invasion in HepG2 cells and the development throughout the liver cycle [57].

The egress process in *Plasmodium* exposes the parasite to a change in the microenvironment. The parasite is exposed to K^+^ concentration, variation from 5 mM in the bloodstream to 140 mM in the host cell cytoplasm. By using schizonts marked with the exogenous Ca^2+^ indicator, Fluo-4/AM, Singh et al. (2010) [58] demonstrated that the transfer of *P. falciparum* merozoites from a high (K^+^) (140 mM) to a low (K^+^) (5 mM) buffer results in cytoplasmatic (Ca^2+^) rise. Moreover, a shift in K^+^ concentration leads to an increase in the expression of the microneme (secretory organelles of parasitic apicomplexans) proteins EBA175 (erythrocyte-binding antigen-175) and AMA-1 (apical membrane antigen-1) on the surface of the parasites [58]. These events are induced in the presence of a Ca^2+^ A23187 ionophore and inhibited after treatment with BAPTA-AM. Even in the absence of extracellular Ca^2+^, the change in (K^+^) induced the same effect, but treatment with PLC inhibitor U73122 abolished the response. The authors proposed that the contact of merozoites with the extracellular environment induces changes in cytoplasmatic (Ca^2+^) via PLC-IP3 and secretion of apical proteins responsible for interaction with the host cell.

*Plasmodium* protein kinase G (PKG) is involved in parasite motility and invasion during the hepatic phase through the activation of CDPK4 (Ca^2+^ dependent protein kinase). Using *P. berghei* parasites mutants for PKG or that carried deletions in the gene encoding CDPK4, Govindasamy et al. (2016) [59] demonstrated that sporozoites from these strains showed inefficiency in the invasion of HepG2 cells. In addition, the treatment of sporozoites with PKG and CDPK4 inhibitors inhibited parasite motility [59]. Protein kinase A is also involved in parasite’s development and can be activated by melatonin in a Ca^2+^ dependent signaling process [60].

In the Anopheles mosquito’s intestine, *Plasmodium* parasites encounter low temperatures, pH differences, and xanthurenic acid. The last is capable of inducing an increase in cytoplasmatic (Ca^2+^) via PLC-IP3 in gametocytes, and the formation of cGMP, regulating Ca^2+^ oscillations through the production of IP3 [61]. Using genetically modified parasites and specific PKG inhibitors, McRobert et al. (2013) [62] demonstrated that in the absence of xanthurenic acid, the increase in cGMP formation by inhibiting phosphodiesterase activates the complete maturation of male and female gametocytes of *P. falciparum*. On the other hand, both in the presence of xanthurenic acid and high cGMP concentrations, treatment with BAPTA-AM inhibits the maturation of the male gametocytes [62]. In another study, *P. berghei* gametocytes resistant to PKG inhibitor treatment showed inhibition of Ca^2+^ oscillation in the presence of xanthurenic acid [63].

*P. falciparum* phosphoproteome revealed that PfCDPK1 may be downstream to GMPc/PKG signaling cascade [63,64] and apical proteins responsible for the invasion and egress of the host cell are PfCDPK1 substrates [65,66]. A recent study showed that PfCDPK1 knockout *P. falciparum* parasites showed deficiency during intra-erythrocytic development, with low growth compared to wild-type parasites, and changes in the expression of AP2-G and GDV1 genes in gametocytes [67]. Furthermore, the PfCDPK1-KO parasites were unable to complete gametogenesis and infect mosquitoes.

In *P. berghei*, the *cpdk3* gene’s deletion results in ookinetes with impaired motility and access to the mosquito’s intestinal epithelium, which decreases the transmissibility of the parasites [68,69]. Sporozoites with deletion of the cdpk3 gene were shown to be viable, which demonstrates that the activity of PbCDPK3 is probably limited to ookinetes [69].

Like CDPK1 and CDPK3, CDKP4 plays an important role during the infection of parasites in mosquitoes. Through the use of inhibitors and the generation of genetically modified parasites, it was possible to determine that, both in *P. berghei* and in *P. falciparum*, after the infection of mosquitoes, CPDK4 participates in the regulation of the parasites’ cell cycle, in the replication of genetic material, as well as in the gametogenesis process [70,71]. In *P. falciparum*, the induction of PfCDPK5 deficiency in schizonts results in merozoites unable to egress, even with the apical complex protein secretion [72,73].

## 3. Mitochondrial Calcium Dynamics and Signaling in Apicomplexan Parasites

Considered the energetic matrix of the cell, the mitochondria are also fundamental in cellular Ca^2+^ homeostasis. After the release of Ca^2+^ through the IP3R and RyR channels present in the ER membrane, these ions can be quickly incorporated by the mitochondria through regions of interaction between the two organelles observed in fungi and different mammalian cells, and where membranes associated with the mitochondria (MAM) interact with the ER network [74,75]. In addition, the evidence points to the existence of a large number of molecules that mediate communication between the ER and the mitochondria for controlling several intracellular signals induced by Ca^2+^ oscillations, for example, during ER stress and control over the generation of reactive oxygen species (ROS) [75,76,77].

Mitochondrial calcium uniporter (MCU) is an essential protein for the transport of calcium across the mitochondrial membrane and has a fundamental role in the regulation of Ca^2+^ signaling, in apoptosis, and in aerobic respiration (for review, see [78]). The first MCU described among less complex life forms, such as plants, invertebrates, insects, and yeasts, was in *Trypanosoma cruzi* [79]. Similar properties to those found in mammalian MCU were observed, such as low sensitivity to Ca^2+^, sensitivity to ruthenium red, and electrogenic transport. The knowledge that *Trypanosoma* and *Leishmania* had a protein that played the role of the MCU was fundamental to the work carried out by De Stefani et al. (2011), who used comparative in silico analysis of conserved sequences to determine the sequence corresponding to the MCU [80].

Several proteins located at the inner and outer mitochondrial membrane play a central role in regulating the absorption and release of Ca^2+^ [81]. The voltage-dependent anion channel (VDAC1), present in the mitochondrial outer membrane, allows the influx of Ca^2+^ into the space between membranes, being fundamental for the decrease of cytoplasmatic (Ca^2+^), and on the other hand, also transports the Ca^2+^ back to the cytoplasm [82]. Free Ca^2+^ ions in the intermembrane space are transported to the mitochondrial matrix via the mitochondrial calcium uniporter (MCU), present in the inner mitochondrial membrane [80]. Na^+^/Ca^2+^ exchange pumps found in the inner membrane allow the Ca^2+^ to go from the mitochondrial matrix to the intermembrane space [81].

**Figure 1:**
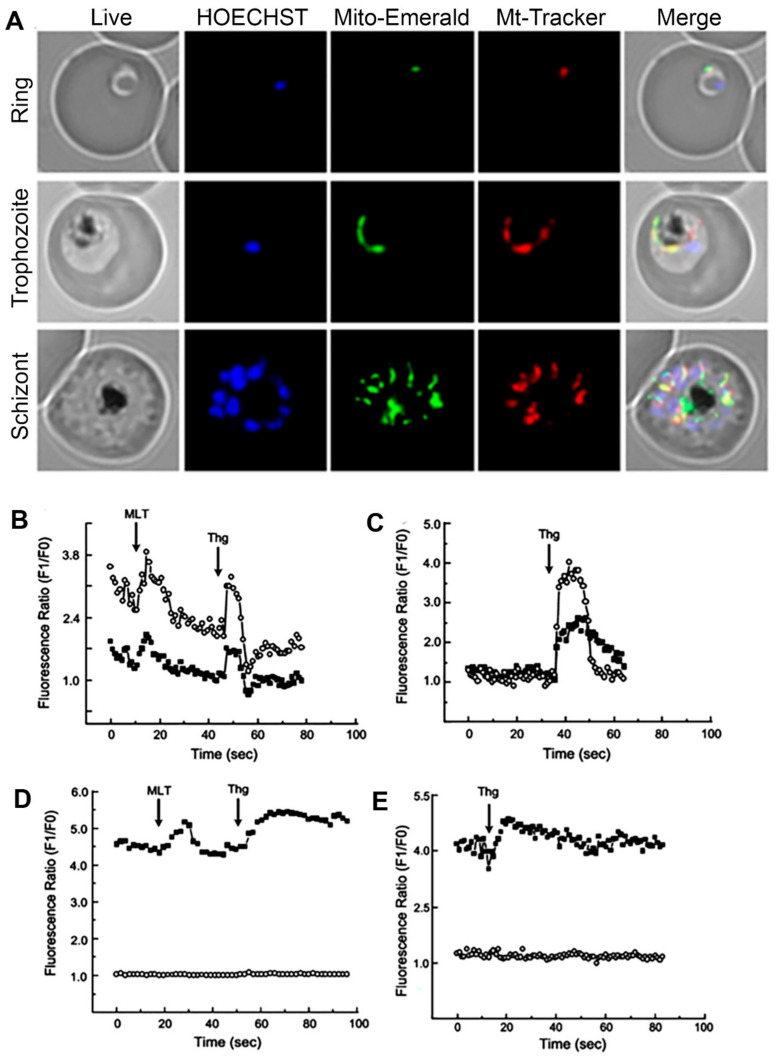
Fluorescent microscopy *Plasmodium falciparum* ring, trophozoites, and schizonts stages using a mitochondrial green fluorescent protein (GFP) construction: the parasite nucleus was stained by HOECHST33342 (blue) and mitochondria with MitoTracker Red CMX Ros (red) to demonstrate co-localization with Mito-Emerald-GFP (green) (**A**). *P. chabaudi* parasites were stained with Fluo4 (cytoplasmatic calcium indicator) and Rhod2 (mitochondrial calcium indicator): effect of melatonin (MLT) and thapsigargin (Thg) on Ca^2+^ (**B**); addition of thapsigargin (**C**); effect of melatonin and thapsigargin on Ca^2+^ fluorescence in the presence of FCCP (**D**); addition of thapsigargin in the presence of FCCP (**E**). Traces represent fluorescence intensity ratio of Ca^2+^ probes Rhod-2 AM-mitochondria (open circles) and Fluo-3 AM-cytosol (fill squares). The images were retrieved with the author’s consent from the following references: [84] (**A**) and [83] (**B**–**E**).

In addition to participating in the reduction of the cytosolic concentration of Ca^2+^, the uptake of this ion by the mitochondria is also fundamental for the ATP synthesis, activating Ca^2+^-dependent enzymes, oxidative phosphorylation, metabolic carriers, and reactive oxygen species (ROS). Ca^2+^ overload of the mitochondrial matrix compromises the functioning of this subcellular compartment. This event results in a decrease in ATP production, an increase in ROS concentration, and induction of the cell death process [75,83,84]. Likewise, in other eukaryotes, *P. chabaudi* and *P. falciparum*’s mitochondria can sequester cytoplasmatic Ca^2+^ during the increase in the concentration of this ion within the cells. This increase may be due to an ER discharge caused by Thg and CPA, or stimulation with an agonist that results in a signaling event and culminates in the release of Ca^2+^ [83] (Figure 1). Of note, the transport of Ca^2+^ into the mitochondria is membrane potential-dependent since the pretreatment with electron transport chain uncoupler Carbonyl cyanide 4-(trifluoromethoxy)phenylhydrazone (FCCP) prevents this process. The action of melatonin on the mitochondrial dynamics of *P. falciparum* has also been reported. This hormone can activate the expression of mitochondrial fission-related genes in a stage-specific manner [84]. Results reported by Rotmann et al. (2010) suggest that a Ca^2+^/H^+^ exchange protein (PfCHA, PF3D7_0603500) may be responsible for the mitochondrial Ca^2+^ efflux in *P. falciparum* [85].

The addition of Ca^2+^ to a buffer containing digitonin permeated *P. berghei* trophozoites causes stimulation of mitochondrial respiration, proportionally to Ca^2+^ concentration in the medium, altering stage 4 of cellular respiration. Oximetry experiments revealed a decrease in mitochondrial membrane potential, proportional to Ca^2+^ concentration added to the buffer, which is compatible with Ca^2+^ influx into the matrix. The addition of succinate to a medium containing digitonin permeabilized trophozoites and 3.5 µM Ca^2+^ led to a drop in this concentration to 0.6 µM. The subsequent addition of FCCP caused a massive increase in free Ca^2+^ in the medium, indicating an efficient mitochondrial calcium transport mechanism is operative [86].

Ca^2+^/H^+^ antiporter (PfCHA) has been described as a mitochondrial transport of divalent ions responsible for exchanging H^+^ for Ca^2+^ or Mn^2+^, with kinetics supporting the role of mitochondria as a dynamic Ca^2+^ stock. Although a homologous protein in humans could not be found, PfCHA has the function of transporting the excess of these ions back to the cytoplasm, helping to maintain mitochondrial concentrations. The low affinity for Ca^2+^ (Tm of 2.2 mM) supports the hypothesis that this transporter would only act in conditions where the mitochondria is loaded with Ca^2+^ [85].

Knowledge of the role of mitochondrial Ca^2+^ in trypanosomes is limited when compared to mammalian cells because Ca^2+^ regulated dehydrogenases are not present or have not been well studied so far. Pyruvate dehydrogenase E1, which is sensitive to Ca^2+^, had its gene identified in *T. cruzi* and has phosphorylation sites to regulate the activity, but there is no evidence that this enzyme works in the same way as in mammals [87]. Mitochondrial isocitrate dehydrogenase is dependent on NADP, unlike the same mammalian enzyme, whose activity depends on NAD and is regulated by Ca^2+^ [88]. There is no evidence of expression of FAD-glycerol phosphate dehydrogenase, which is activated by Ca^2+^ in animals, in trypanosomes [89]. Moreover, the aspartate-glutamate and ATP-Mg-Pi carriers, which in mammals are regulated by Ca^2+^, are present in trypanosomes but do not have the EF-hand domain for binding to Ca^2+^ and are probably insensitive to this ion [90].

Similar to mammals, Trypanosomatids have a well-characterized MCU-mediated Ca^2+^ influx mechanism, although some proteins are absent [91,92]. Interference in MCU expression using siRNA or conditional knockdown causes a deficiency in the influx of mitochondrial Ca^2+^. It directly reflects in the metabolism, resulting in abnormal ATP concentrations, growth defects, and autophagy. These effects become more prominent when oxidative phosphorylation is essential, such as in low-glucose media found in the insect vector. On the other hand, MCU overexpression results in a pro-apoptotic state with Ca^2+^ overloaded mitochondria [91]. Other studies with T. cruzi supported these findings: using a CRISPR/Cas9 knockout system, there is a deficiency in calcium influx without changing the mitochondrial membrane potential. Although the parasites remain viable, the growth defects are notable: low cellular respiration, increased autophagy, and low infectivity [93,94].

As observed in *Plasmodium*, trypanosome mitochondria are also capable of acting as a Ca^2+^ buffer. The uptake of cytoplasmic Ca^2+^ into the mitochondria can occur in both molar and micromolar concentrations, depending on the membrane potential [95]. This phenomenon suggests an approximation of mitochondria with microdomains of high Ca^2+^ concentration, such as plasma membrane, acidocalcisomes, or endoplasmic reticulum [96]. As IP3R has not been identified, and SERCA has low sensitivity to Thg, a link between the transport of calcium from the endoplasmic reticulum to the mitochondria has not yet been established [97]. Despite this, it is expected that the MCU will act as a modulator of the cytoplasmic Ca^2+^ space-temporal fluctuations resulting from various cellular processes [98,99]. Another important role that mitochondrial Ca^2+^ is its involvement with apoptosis. This process in trypanosomes is well studied, although some important effector and regulatory molecules have not yet been described, such as caspases and TNF receptors [100,101]. In *T. brucei*, the production of reactive oxygen species blocks the mitochondrial transport of Ca^2+^, which results in its accumulation in the nucleus and causes cell death [102]. In *T. cruzi*, calcium overload-related apoptosis is dependent on the formation of superoxide ions [103].

Elmahallawy et al. (2014) investigated the harmful effects of melatonin in *Leishmania infantum* promastigotes. Using concentrations between 25 and 50 nM, significant inhibition of complexes I, III, and IV of the electron transport chain was noted, and eventually, the death of the parasite [104]. In addition, the authors identified that melatonin caused changes in Ca^2+^ homeostasis by altering the functioning of the mitochondrial permeability transition pore, controlling the capacity of mitochondrial Ca^2+^ retention and release, which can also be associated with cell death [105]. 

Apicomplexa organisms have peculiar and unusual mitochondria, with primitive characteristics that place the organisms of this group as strong candidates to the first existing eukaryotes. Among these characteristics, the presence of a single mitochondria during most of the life cycle, a small and highly fragmented mitochondrial genome, simple protein import machinery, and low activity of the electron transport chain stand out, all of this in organisms with a need to adapt to several different microenvironments. In addition to the obvious public health need to study these organisms in order to identify new therapeutic targets, the study of parasites’ biology can help to understand molecular aspects still unknown in more complex organisms, such as mammals.
